# Trapezoidal Subwavelength Grating Micro-Ring Resonator with Envelope-Spectrum Tracking for Ultra-Wide-Range Refractive Index Sensing

**DOI:** 10.3390/s26206502

**Published:** 2026-10-14

**Authors:** Hongjun Gu, Junhan Chang, Jintao Su, Meiling Zhang, Xue Li, Jing Zhou, Lixin Hou

**Affiliations:** 1 College of Information Technology, Jilin Agricultural University, Changchun 130118, China; guhongjun@jlau.edu.cn (H.G.); 20241252@mails.jlau.edu.cn (J.C.); 20261425@mails.jlau.edu.cn (J.S.); lixue@jlau.edu.cn (X.L.); 012028@jlau.edu.cn (J.Z.); 2 Department of Communication Engineering, Jilin University, Changchun 130012, China; zhangmeiling@jlu.edu.cn

**Keywords:** micro-ring resonator, subwavelength grating, ultra-high sensitivity, ultra-wide detection range

## Abstract

This paper proposes a trapezoidal subwavelength grating micro-ring resonator refractive index sensor that integrates subwavelength grating structures with a micro-ring resonator to achieve highly efficient mode conversion, ultra-high sensitivity, and an ultra-wide detection range. The proposed device is numerically investigated using the 2.5-dimensional finite-difference time-domain method. The mode converters, including strip-to-slot, slot-to-double-slot, and double-slot-to-SWG, exhibit a simulated average mode conversion efficiency exceeding 95%. The double-slot structure enhances the light-analyte interaction, thereby improving the sensing sensitivity, while the trapezoidal SWGs in the rings further improve sensing performance. Simulations yield an RI sensitivity of 1127 nm/RIU and a concentration sensitivity of 2780 pm/%. Moreover, the simulated detection range of the TSWG-MRR is extended to 0.45 RIU, overcoming the free spectral range limitation of conventional MRRs. The proposed TSWG-MRR achieves a quality factor of 1885, an extinction ratio of 15.7 dB, and a theoretically calculated limit of detection of 8.87 × 10^−6^ RIU and demonstrates strong potential for lab-on-a-chip sensing applications.

## 1. Introduction

With the rapid development of biomedical diagnostics, environmental monitoring, food safety inspection, and chemical analysis, there is an increasing demand for sensing technologies capable of providing high sensitivity, a wide detection range, real-time response, and label-free detection [1,2,3,4]. Among the various sensing approaches, optical refractive index (RI) sensors have attracted significant attention owing to their immunity to electromagnetic interference, high measurement accuracy, compact footprint, and excellent compatibility with photonic integrated circuits. By monitoring variations in optical parameters induced by interactions between guided optical fields and surrounding analytes, RI sensors enable highly sensitive and quantitative detection of chemical and biological substances.

Among the numerous integrated photonic sensing platforms, micro-ring resonators (MRR) have emerged as one of the most promising candidates due to their compact size, high quality factor (Q), low power consumption, and compatibility with mature silicon-on-insulator (SOI) fabrication technologies [5,6]. Conventional MRR-based sensors typically operate by tracking resonance wavelength shifts caused by changes in the surrounding RI [7,8]. However, their performance is fundamentally constrained by two major limitations. First, the interaction between the evanescent optical field and the analyte is relatively weak, which restricts the achievable sensitivity. Second, the measurable sensing range is limited by the free spectral range (FSR) of the MRR, thereby hindering applications requiring wide-range RI measurements. Consequently, simultaneously achieving ultra-high sensitivity and a broad sensing range remains a critical challenge for MRR-based sensing systems.

To enhance the sensitivity of MRR sensors, considerable efforts have been devoted to strengthening light–matter interactions through structural engineering. Representative approaches include the incorporation of slot waveguides into MRRs [9,10,11,12,13], cascaded resonator configurations [14,15,16], and hybrid structures integrating Mach–Zehnder interferometers (MZIs) with MRRs [17,18]. Slot-waveguide-based resonators can strongly confine the optical field within low-index regions, thereby significantly increasing the overlap between the guided mode and the surrounding analyte. Claes et al. reported a slot-waveguide MRR with a sensitivity of approximately 398 nm/RIU [19]. The cascaded resonator structure has achieved sensitivity of up to 489.3 nm/RIU [16], while our previously reported MZI-assisted slot MRR demonstrated a sensitivity as high as 1009 nm/RIU [17]. Despite these substantial improvements in sensitivity, the corresponding sensing ranges generally remain limited to 0.01–0.07 RIU, which restricts their applicability in wide-range sensing scenarios [16].

Recently, subwavelength grating (SWG) waveguides have emerged as a promising approach for enhancing the sensing performance of integrated photonic devices. In SWG waveguides, periodic subwavelength modulation engineers the effective refractive index and dispersion of the guided mode, which benefits sensing in two distinct aspects. On the one hand, the engineered field distribution delocalizes the optical mode into the low-index analyte region, strengthening the evanescent light–matter interaction and hence the sensitivity [20,21,22]. On the other hand, the dispersion property of the SWG waveguide offers a route to extend the measurable sensing range, which is defined in this work as the maximum ambient refractive index change that can be tracked unambiguously. In conventional MRRs, sensing relies on monitoring the wavelength shift of an individual resonance peak; once the shift exceeds the FSR, the shifted peak can no longer be unambiguously assigned to a specific resonance order, so the measurable sensing range is fundamentally bounded by the FSR [23]. To overcome this FSR-imposed limit, Xu et al. proposed tracking the envelope spectrum of a dispersive SWG-MRR rather than a single resonance peak; because the envelope evolves continuously with the ambient RI, the unambiguous tracking range is liberated from the FSR restriction [24], and dispersion-engineered designs have further demonstrated high sensitivity together with an extended range [25]. Following these concepts, Huang et al. developed an SWG racetrack resonator with a sensing range of 0.11 RIU and a sensitivity of 429 nm/RIU [20], and Kazanskiy et al. further combined slot waveguides with SWG structures to achieve a sensitivity of 760 nm/RIU [21]. Nevertheless, the corresponding sensing ranges remained below 0.1 RIU, which is still insufficient for applications requiring large-scale RI monitoring. These studies indicate that although the envelope-spectrum concept has partially alleviated the FSR-imposed sensing-range limitation, achieving both ultra-high sensitivity and ultra-wide sensing range simultaneously remains a significant challenge.

To address this issue, we propose a trapezoidal subwavelength grating micro-ring resonator (TSWG-MRR) featuring a double-slot architecture and an envelope-spectrum tracking mechanism. Unlike conventional MRR sensors that rely on monitoring the shift of a single resonance peak, the proposed device utilizes the global displacement of an SWG-induced envelope spectrum as the sensing signature. By tracking the movement of the entire envelope spectrum in response to RI variations, the proposed sensing strategy effectively overcomes the intrinsic FSR limitation while maintaining ultra-high sensitivity.

The TSWG-MRR incorporates two major innovations. First, at the structural level, a double-slot SWG architecture combined with trapezoidal silicon blocks is introduced into the MRR. The double-slot configuration significantly enhances the interaction between the optical field and the surrounding analyte, while the trapezoidal geometry improves optical confinement, suppresses scattering loss, and promotes efficient optical propagation within the slot region [22,26,27]. Second, at the sensing-mechanism level, the strong wavelength-dependent dispersion characteristics of the SWG structure are exploited to generate a distinct envelope-shaped transmission spectrum. Consequently, sensing can be performed by tracking the global envelope shift rather than individual resonance peaks, thereby substantially extending the measurable RI range [28,29].

To validate the proposed concept, comprehensive numerical simulations are conducted using the 2.5D finite-difference time-domain (FDTD) method. Key structural parameters are systematically optimized to maximize sensing performance. Furthermore, high-efficiency mode converters, including strip-to-slot (STS), slot-to-double-slot (STDS), and double-slot-to-SWG (DSTS) converters, are designed to ensure low-loss optical transitions between conventional strip waveguides and the SWG sensing structure.

The optimized TSWG-MRR achieves an RI sensitivity of 1127 nm/RIU, a concentration sensitivity of 2780 pm/%, an extinction ratio (ER) of 15.7 dB, and a Q of 1885. More importantly, the proposed envelope-spectrum tracking strategy extends the sensing range to 0.45 RIU, which is more than one order of magnitude larger than that of conventional MRR-based sensors. These results demonstrate that the TSWG-MRR effectively mitigates the longstanding trade-off between sensitivity and sensing range, providing a promising platform for next-generation integrated photonic sensing systems and lab-on-a-chip applications.

## 2. Structure Design and Principle

**Figure 1:**
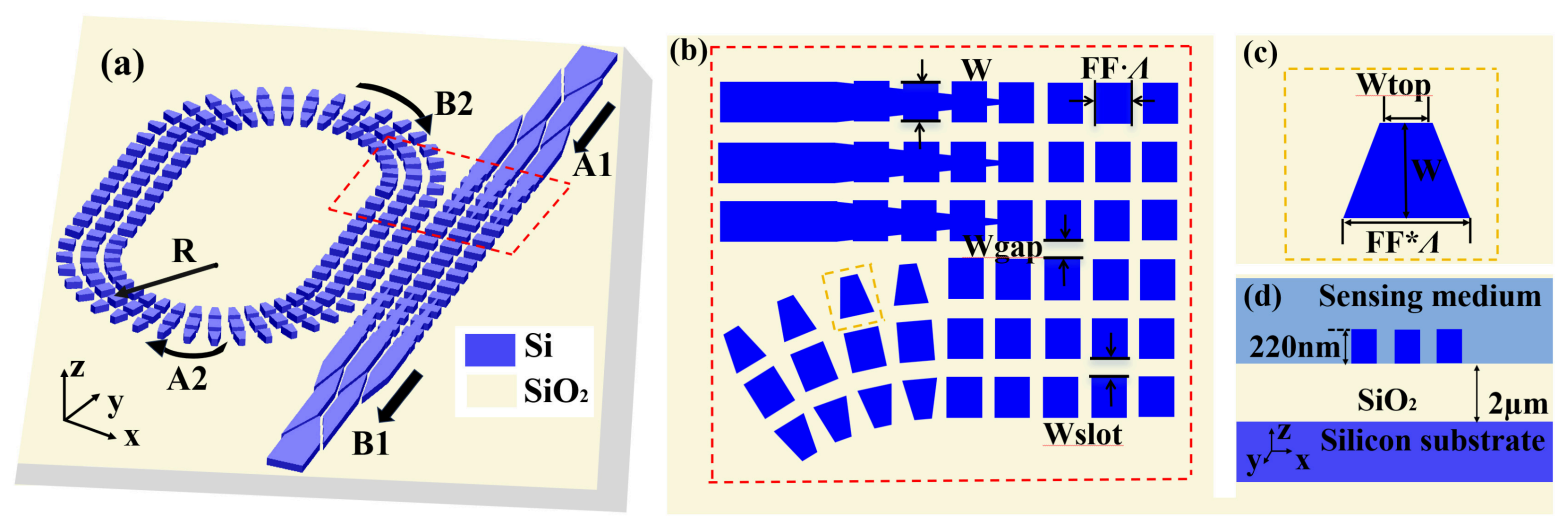
(**a**) Schematic diagram of the TSWG-MRR on the SOI platform; (**b**) schematic diagram of the coupling section; (**c**) schematic diagram of a trapezoidal structural unit; (**d**) cross-sectional view of the waveguide.

The TSWG-MRR is implemented on a commercially available SOI platform featuring a 220 nm-thick silicon device layer and a 2 μm buried oxide layer. The overall structure of the sensor is illustrated in Figure 1a. Both the bus waveguide and the racetrack resonator are constructed using three parallel rows of SWGs arranged in a double-slot configuration, enabling efficient optical confinement and enhanced interaction between the optical field and the surrounding analyte. A1 and B2 denote the input optical amplitudes, and A2 and B1 represent the corresponding output amplitudes of the coupling region. Optical power is coupled between the bus waveguide and the resonator through evanescent-field interaction across the coupling distance. Variations of the surrounding refractive index modify the effective RI of the guided mode, thereby shifting the resonance condition of the MRR and producing measurable spectral changes.

The geometrical parameters of the TSWG-MRR are defined in Figure 1b–d. The SWG waveguide consists of periodically arranged silicon blocks with width W and length FF·*Λ*, where FF denotes the fill factor of the subwavelength grating and *Λ* represents the grating period. The slot width between adjacent SWG waveguides is denoted by Wslot, while the coupling gap between the bus waveguide and the racetrack resonator is represented by Wgap. For the trapezoidal silicon blocks located in the curved sections of the resonator, the width of the top base of the trapezoidal silicon block is denoted by Wtop, and the lower width corresponds to FF·*Λ*. The bending radius is expressed as R.

Unlike conventional SWG resonators employing rectangular silicon segments, the curved sections of the proposed racetrack resonator adopt trapezoidal silicon blocks, as illustrated in Figure 1c. The trapezoidal geometry gradually modifies the effective RI distribution along the propagation direction, thereby reducing scattering losses caused by abrupt geometric discontinuities. Furthermore, the trapezoidal profile improves optical-field confinement within the slot regions and increases the overlap between the evanescent field and the surrounding sensing medium. As a result, the proposed structure simultaneously enhances the sensitivity and optical transmission performance.

The periodic SWG structure supports Floquet–Bloch propagation modes when the grating period is substantially smaller than the operating wavelength. Under this subwavelength condition, diffraction effects are effectively suppressed, allowing the periodic waveguide to behave as a homogeneous medium with engineered dispersion properties [30]. Consequently, the SWG waveguide can guide optical modes in a manner similar to conventional strip waveguides while providing additional flexibility for tailoring modal confinement and dispersion characteristics.

### 2.1. High-Efficiency Mode Converter Design

**Figure 2:**
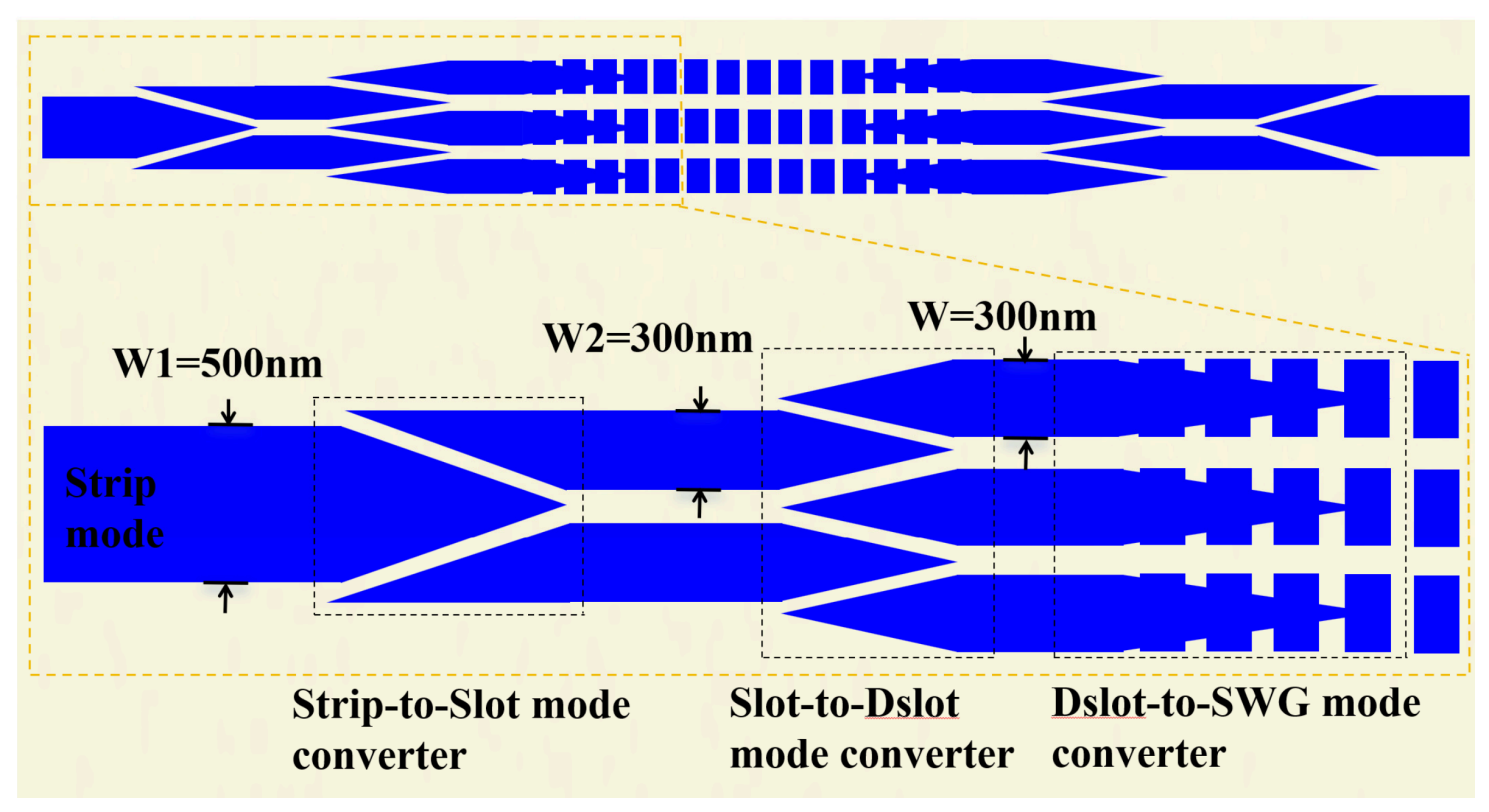
Schematic diagram of the strip waveguide to SWG waveguide structure.

**Figure 3:**
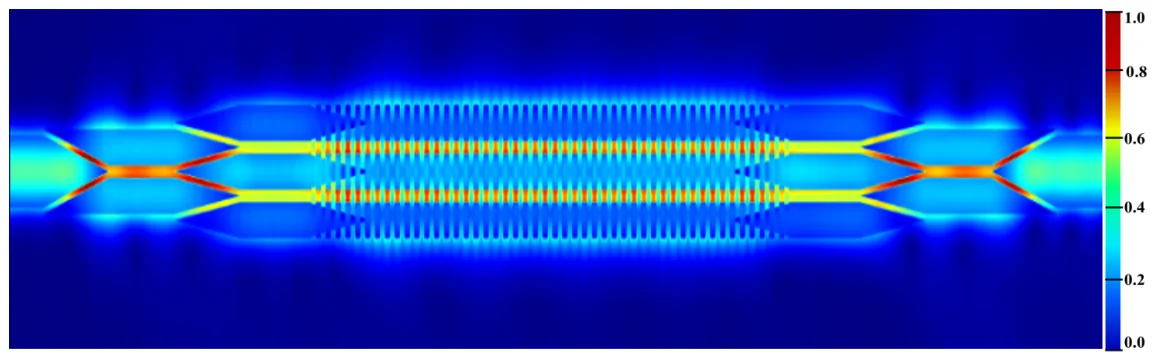
The electric field distribution of the converter.

**Figure 4:**
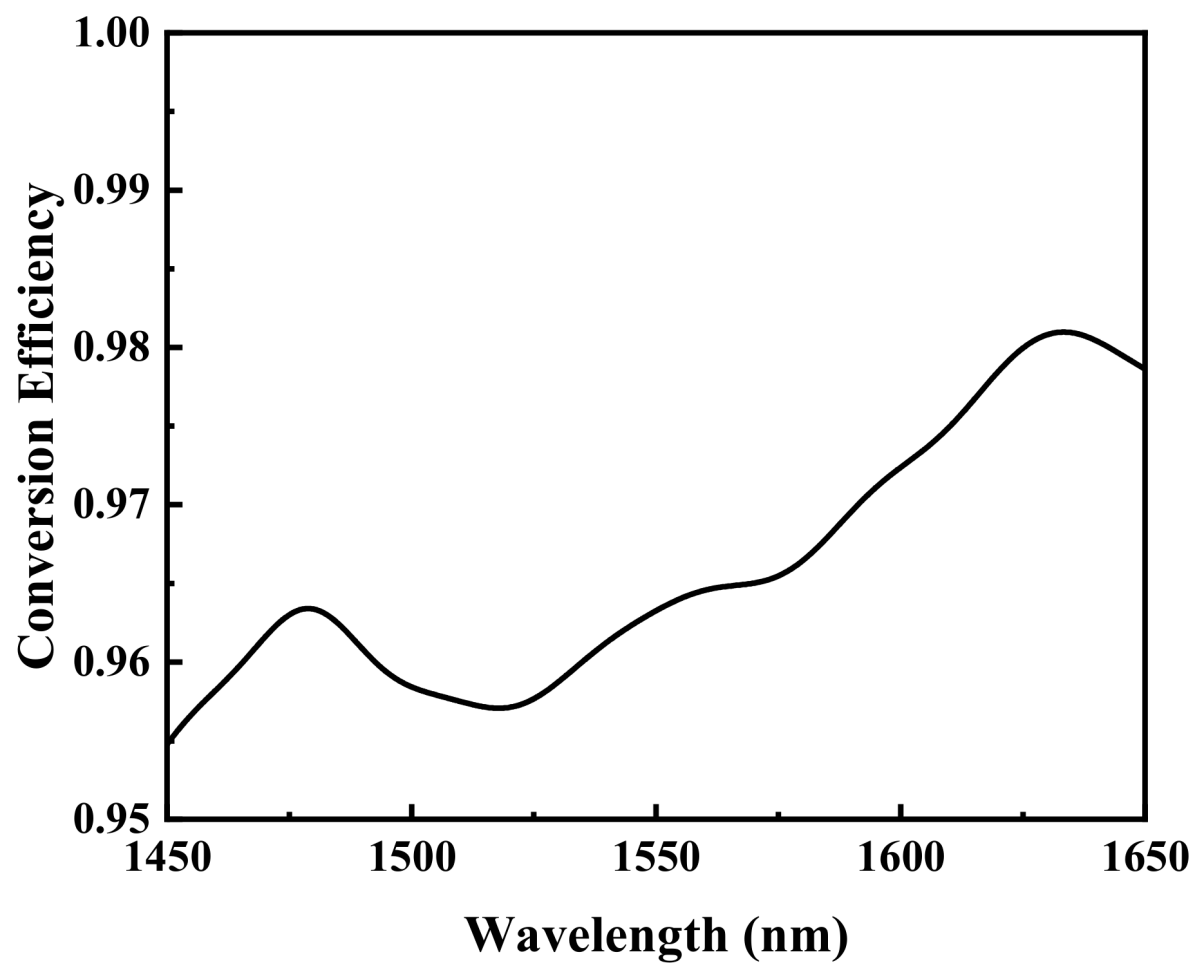
Total conversion efficiency of the mode converter versus wavelength.

Direct coupling between a conventional strip waveguide and a double-slot SWG waveguide results in significant mode mismatch and insertion loss. To overcome this issue, a multi-stage mode-conversion system is introduced, as shown in Figure 2. The proposed mode converter consists of three sequential sections: an STS converter, STDS converter, and DSTS converter. The strip waveguide W1 is 500 nm wide, and the slot-waveguide W2 and double-slot waveguide W share the same width of 300 nm. The STS section gradually transforms the optical mode supported by a conventional strip waveguide into a slot-waveguide mode. Subsequently, the STDS section converts the single-slot mode into a double-slot mode while maintaining high modal overlap and low propagation loss. Finally, the DSTS section employs tapered waveguides with gradually varying widths to achieve efficient coupling between the double-slot waveguide and the SWG structure. This cascaded conversion strategy significantly improves mode matching between the fundamental strip-waveguide mode and the Bloch mode supported by the SWG waveguide. Consequently, coupling loss is substantially reduced, enabling efficient optical power transfer throughout the sensing structure. The simulated electric-field distribution of the mode converter is shown in Figure 3. The optical field remains strongly confined within the slot regions during the entire conversion process, indicating excellent modal continuity and negligible radiation loss. As depicted in Figure 4, the corresponding wavelength-dependent mode converter achieves a conversion efficiency of over 95% across the wavelength range of 1450–1650 nm, covering the S-, C-, and L-bands, demonstrating excellent broadband performance and low insertion loss.

To evaluate the mode-conversion performance, the fundamental TE mode (TE0) is launched at the input strip waveguide. The mode-conversion efficiency is defined as the ratio of the transmitted optical power at the output of the DSTS section to the power of the launched mode, i.e., η = P_out_/P_in_, and the corresponding coupling loss is calculated as L = −10·lg(η) in dB. Both quantities are extracted from the 2.5D FDTD simulations at the output monitoring plane, where the mode has evolved into the Bloch mode supported by the double-slot SWG waveguide.

### 2.2. Theoretical Analysis of TSWG-MRR

The amplitude coupling coefficient and amplitude transmission coefficient of the coupling region are denoted as *k* and *t*, respectively. Let *α*_0_ be the optical field propagation loss coefficient in the ring waveguide, which accounts mainly for the scattering and leakage losses of the SWG sections; the bend radiation at R is negligible and consistent with the lossless coupling condition *|k*(*λ*)|^2^ + *|t*(*λ*)|^2^ = 1. Then, the round-trip amplitude transfer factor *a*(*λ*) can be expressed as Equation (1).

*a*(λ) = e^−α_0_L^ (1)

where *L* represents the perimeter of the ring. Correspondingly, the relationship between the optical fields inside the ring can be expressed as Equation (2).

(2) $$\begin{array}{c} A_{2}=a\left(\lambda \right)e^{-\mathrm{j\beta L}}B_{2} \end{array}$$

where *β* denotes the longitudinal propagation constant. Correspondingly, the round-trip phase factor *φ*(*λ*) of the ring is expressed as Equation (3).

(3) $$\begin{array}{c} \varphi \left(\lambda \right)=\mathrm{\beta L} \end{array}$$

Considering the lossless coupling condition, i.e., *|k*(*λ*)|^2^ + *|t*(*λ*)|^2^ = 1, the optical field equations in the coupling region are given as Equation (4) and Equation (5), respectively.

B_1_ = *t*(*λ*)A_1_ − *ik*(*λ*)A_2_ (4)

B_2_ = *t*(*λ*)A_2_ + *ik*(*λ*)A_1_ (5)

The incident field A_1_ is normalized to unity (A_1_ = 1). Combining the coupling-region equations (Equations (4) and (5)) with the round-trip field relation (Equation (2)) gives B_2_ = kA_1_ + tA_2_ = k + t·a(λ)·e^−jφ^·B_2_, from which B_2_ = k/[1 − t·a(λ)·e^−jφ^]. The through-port field is B_1_ = tA_1_ + kA_2_ = t + k·a(λ)·e^−jφ^·B_2_. Substituting B_2_ and applying the lossless coupling condition |k(λ)|^2^ + |t(λ)|^2^ = 1 yields B_1_ = [t − a(λ)·e^−jφ^]/[1 − t·a(λ)·e^−jφ^], and the transmission intensity T = |B_1_|^2^ directly gives Equation (6), where φ = φ(λ) = βL from Equation (3).

When incident light is injected into one port of the strip waveguide and propagates through the TSWG-MRR, the spatial periodic RI modulation of the SWG induces a dispersion relation characterized by a photonic bandgap or slow-light propagation. Therefore, the transfer function can be described using a dispersion-dependent formulation [24]. The transfer function *T*(*λ*) of TSWG-MRR can be obtained as Equation (6).

(6) $$T(\lambda )={\left|\frac{B_{1}}{A_{1}}\right|}^{2}=\frac{a{\left(\lambda \right)}^{2}+t{\left(\lambda \right)}^{2}-2a(\lambda )t(\lambda )\cos\varphi (\lambda )}{1+a{\left(\lambda \right)}^{2}t{\left(\lambda \right)}^{2}-2a(\lambda )t(\lambda )\cos\varphi (\lambda )}$$

In (6), when *φ*(*λ*) is an integer multiple of 2π, i.e., the light meets the resonance condition, then Equation (6) can be converted into Equation (7).

(7) $$T(\lambda )={\left[\frac{a(\lambda )-t(\lambda )}{1-a(\lambda )t(\lambda )}\right]}^{2}$$

Light whose round-trip optical length in an MRR is an integer multiple of its wavelength can resonate within the ring. When *a*(*λ*) is numerically equal to *t*(*λ*), the device reaches the critical coupling state. The resonance equation is shown in Equation (8).

For the race-track geometry adopted in this work, L denotes the total perimeter of the microresonator, i.e., L = 2πR + 2L_s, where L_s is the coupling length, giving a total perimeter L = 2πR + 2L_s.

(8) $$\begin{array}{c} Ln_{\mathrm{eff}}=m\lambda_{\mathrm{res}},m=1,2,3\ldots \ldots \end{array}$$

Here, n_eff_ is the effective RI, λ_res_ is the resonant wavelength, and *m* stands for the resonance order.

For label-free sensors in practical implementation, sensitivity serves as a key performance indicator. The Q characterizes the energy storage capability of an MRR and directly reflects the sharpness of the resonance peak, and a high ER can enhance the signal-to-noise ratio and reduce noise effects caused by environmental interferences. Herein, *λ_res_* is the full width at half maximum (FWHM) of the resonance peak. Q is the ratio of *λ_res_* to *λ_FWHM_*, and ER is defined as the ratio of the maximum transmission intensity (*I_max_*) to the minimum transmission intensity (*I_min_*) in the transmission spectrum. The mathematical definitions of Q and ER are shown in Equation (9) and Equation (10), respectively.

(9) $$Q=\frac{\lambda_{\mathrm{res}}}{\lambda_{\mathrm{FWHM}}}$$

(10) $$\mathrm{ER}=10\mathrm{lg}\left(\frac{\left|I_{\max}\right|}{\left|I_{\min}\right|}\right)$$

The sensing performance of the designed sensor is tested by varying the ambient RI to simulate changes in the concentration of the sensing medium. The RI sensitivity is defined as Equation (11).

(11) $$S_{n}=\frac{\Delta \lambda }{\Delta n}$$

where Δ*λ* denotes the resonant wavelength shift, and Δ*n* represents the change in the RI of the sensing medium.

## 3. Optimization Process

To maximize the sensing performance of the proposed TSWG-MRR, comprehensive numerical optimizations are carried out using the 2.5D FDTD method implemented in Ansys MODE Solutions. Compared with full three-dimensional simulations, the 2.5D FDTD approach provides comparable accuracy while significantly reducing computational complexity and simulation time. Consequently, the geometrical parameters of the SWG structure strongly influence the modal distribution, optical confinement, and effective RI modulation [31]. Appropriate parameter selection is essential for simultaneously achieving high sensitivity, high Q, and large ER. The FF determines the relative proportion of silicon and low-index regions within each SWG period and plays a critical role in controlling the effective RI and Bloch-mode characteristics of the waveguide.

**Figure 5:**
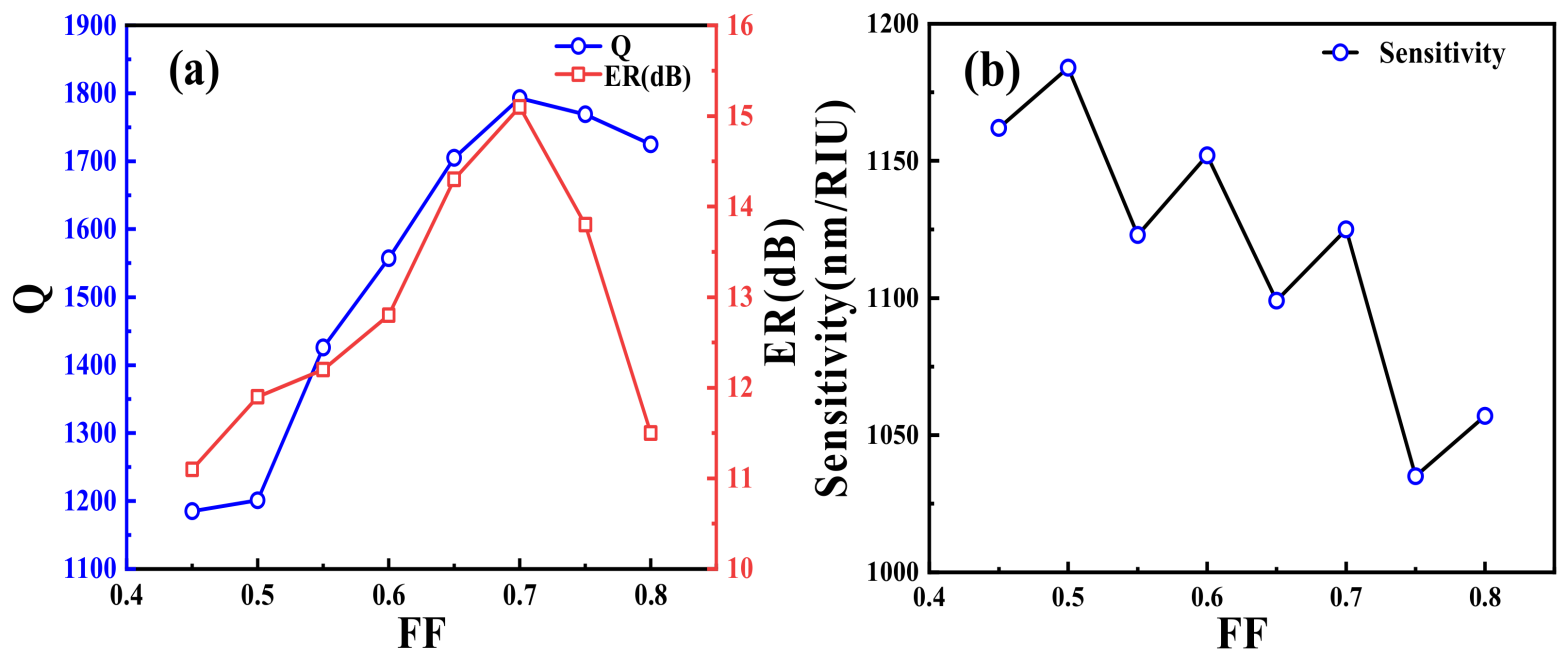
Effect of FF on TSWG-MRR performance: (**a**) variation of Q and ER with FF; (**b**) variation of sensitivity with FF.

Figure 5a illustrates the variations of the Q and ER as functions of FF. Q and ER initially rise with the increase in FF, reach maximum values at FF = 0.7, and subsequently fall. When the FF is too small, the optical field becomes insufficiently confined within the waveguide, resulting in enlarged propagation loss and degraded resonator performance. Conversely, excessively large FF weakens the RI contrast within the SWG structure and reduces the effectiveness of Bloch-mode modulation, thereby deteriorating resonance characteristics. The influence of FF on sensitivity is presented in Figure 5b. As the FF increases, the sensitivity exhibits an overall decreasing trend. This behavior can be attributed to the reduction in the analyte-accessible region within the SWG structure. A larger silicon fraction decreases the overlap between the evanescent field and the surrounding medium, thereby weakening light–matter interaction. Considering the trade-off between sensitivity and Q, FF = 0.7 is selected as the optimal value because it provides relatively high overall performance in terms of Q, ER, and sensitivity.

**Figure 6:**
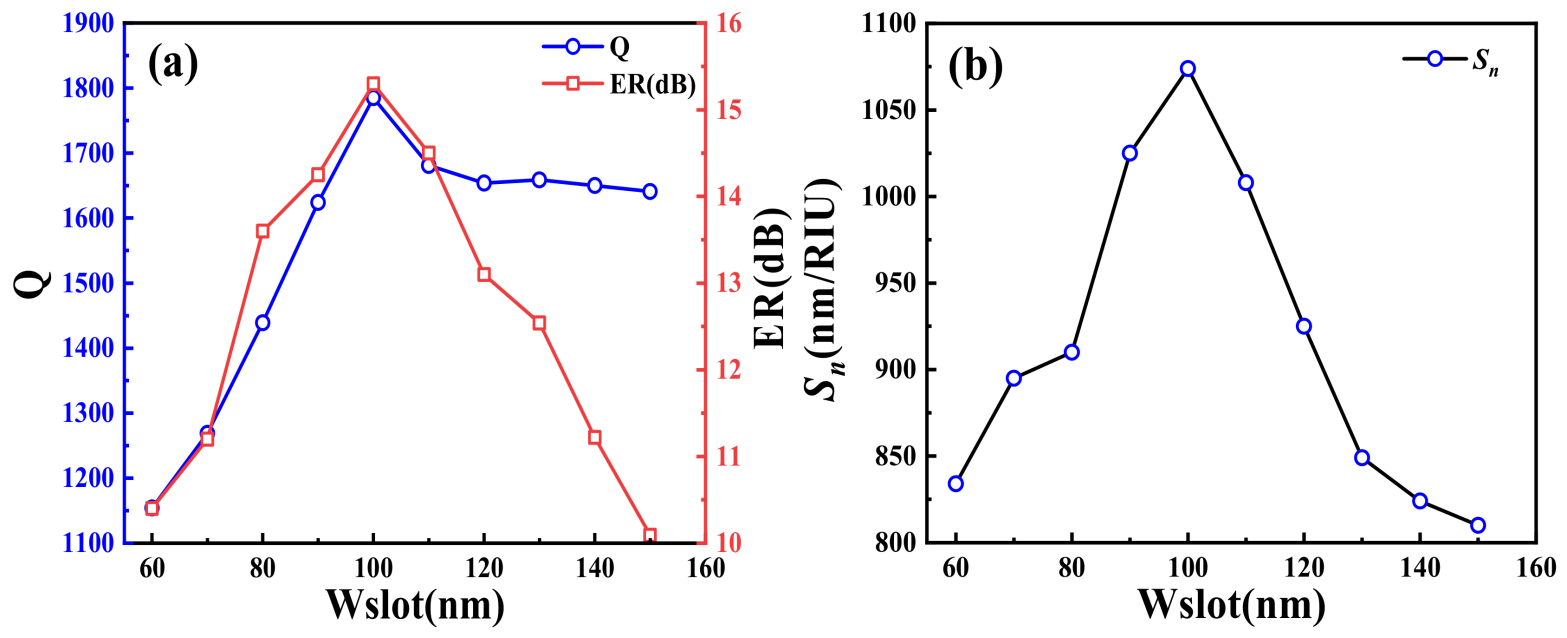
Impact of Wslot on TSWG-MRR performance: (**a**) variation of Q and ER with Wslot; (**b**) variation of sensitivity with Wslot.

The slot width Wslot directly influences the optical-field distribution within the sensing region and plays a key role in determining sensing performance. As shown in Figure 6a, both Q and ER rise with the increase in Wslot and reach their maximum values at approximately Wslot = 100 nm. A narrow slot strongly confines the optical field but limits analyte penetration into the sensing region. The corresponding sensitivity variation is illustrated in Figure 6b. The sensitivity initially increases and reaches its maximum at Wslot = 100 nm, after which it gradually decreases. When the slot becomes excessively wide, optical confinement weakens, and the effective RI contrast decreases, resulting in reduced sensitivity to environmental RI changes. Considering the combined performance metrics, Wslot = 100 nm is selected as the optimal slot width.

**Figure 7:**
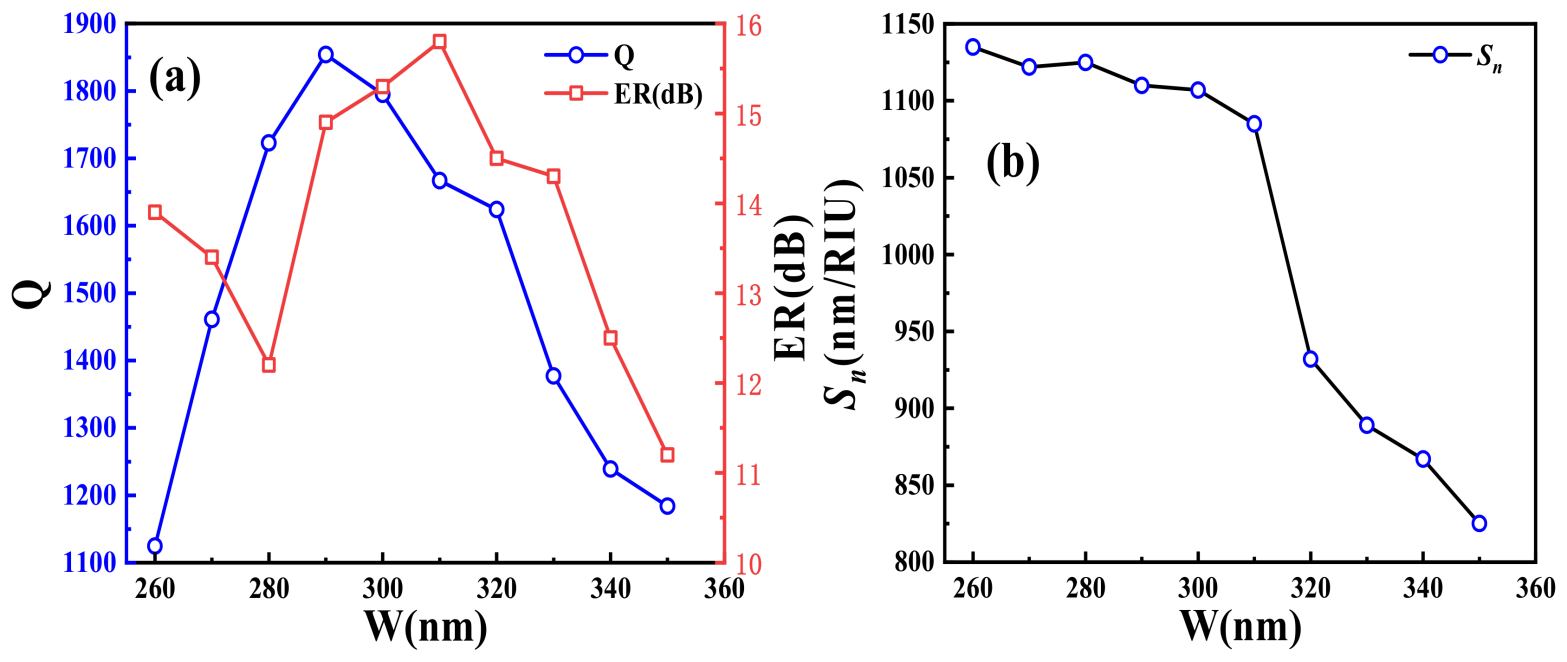
Influence of W on the performance of TSWG-MRR: (**a**) variation of Q and ER with W; (**b**) variation of sensitivity with W.

The grating width W influences both the effective optical path length and the modal confinement characteristics of the SWG waveguide. The variations of W modify the propagation constant and affect the resonance condition of the MRR. As shown in Figure 7a, the Q initially increases with the increase in grating width and reaches its maximum value near W = 290 nm, and ER obtains its maximum at W = 310 nm. Figure 7b demonstrates that the sensitivity remains stable for widths below 300 nm but decreases significantly when W is bigger than this value. Physically, increasing the waveguide width enhances optical confinement and reduces radiation loss. However, excessive confinement reduces the fraction of optical energy residing in the evanescent field, thereby weakening the interaction between light and the surrounding analyte. Consequently, a compromise between Q, ER, and sensing performance must be achieved. Based on these considerations, W = 300 nm is selected as the optimal grating width.

**Figure 8:**
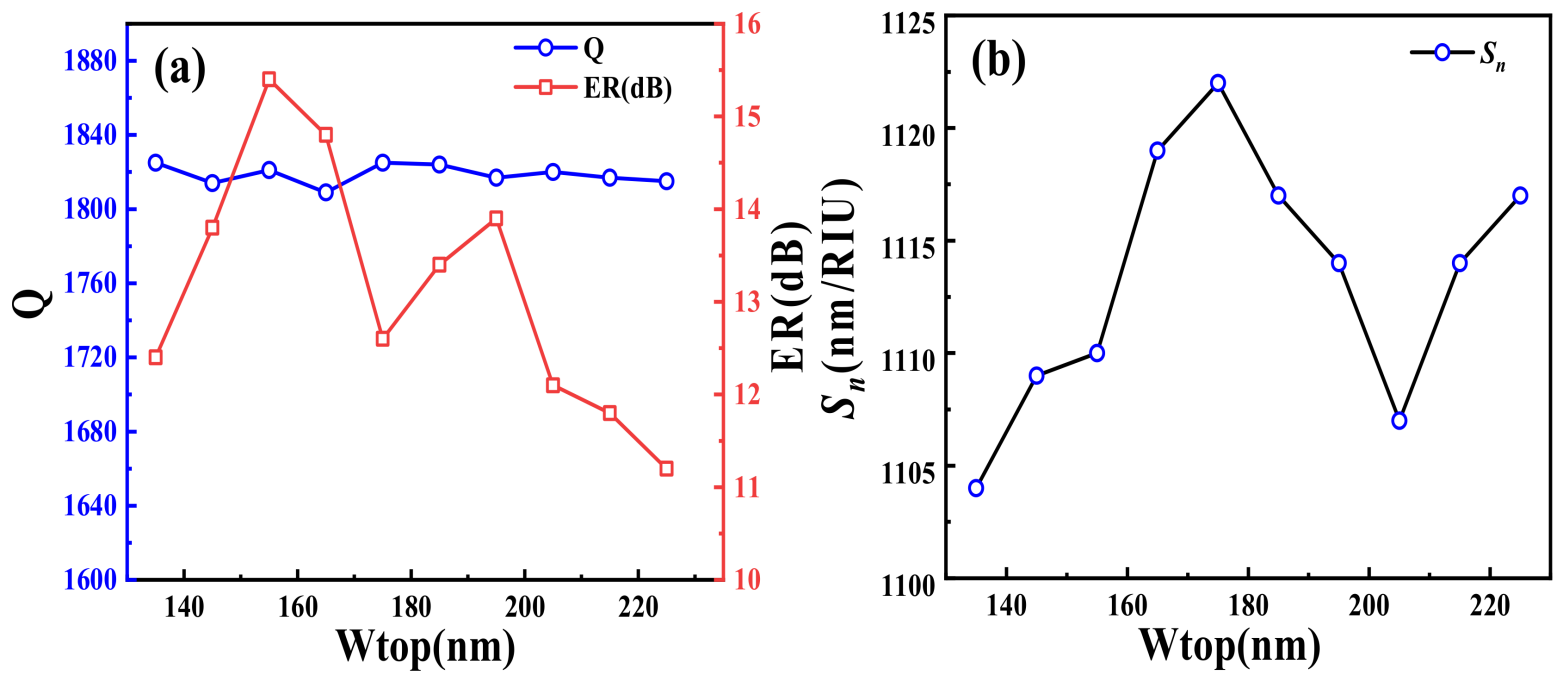
Effect of Wtop on TSWG-MRR performance: (**a**) variation of Q and ER with Wtop; (**b**) variation of sensitivity with Wtop.

To further improve sensing performance, trapezoidal silicon blocks are introduced into the curved sections of the resonator. The Wtop determines the geometrical profile of the SWG element and influences both optical confinement and modal dispersion. Figure 8a presents the variations of Q and ER with different values of Wtop. The Q remains relatively stable throughout the investigated range, whereas the ER exhibits a pronounced maximum at Wtop = 155 nm. The corresponding sensitivity variation is shown in Figure 8b. The sensitivity initially increases and subsequently decreases as Wtop increases. A moderate trapezoidal profile effectively redistributes the optical field toward the slot region and enhances the overlap between the evanescent field and the analyte. However, excessively large top widths increase optical confinement within the silicon region and reduce sensing efficiency. Considering the overall performance, Wtop is determined to be 155 nm to achieve the optimal effect.

**Figure 9:**
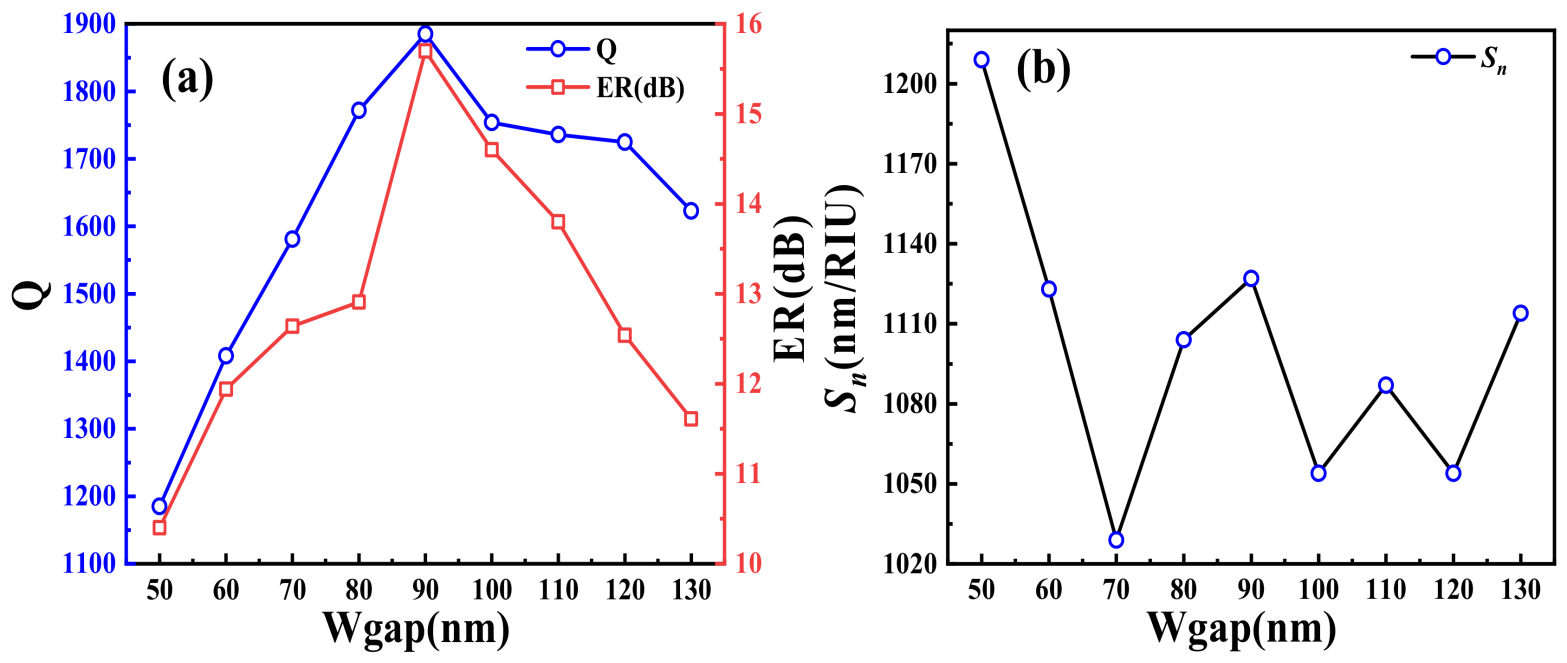
Effect of Wgap on TSWG-MRR performance: (**a**) variation of Q and ER with Wgap; (**b**) variation of sensitivity with Wgap.

The coupling distance Wgap controls the coupling optical intensity between the bus waveguide and the ring and strongly influences the resonance characteristics of the device. As illustrated in Figure 9a, both the Q and ER achieve their maximum values at Wgap = 90 nm. This indicates that the MRR operates close to the critical-coupling condition at this point, enabling efficient power transfer between the bus waveguide and the ring. Figure 9b presents the sensitivity variation with coupling distance. Although the sensitivity exhibits a fluctuating trend, relatively high sensitivity is maintained near Wgap = 90 nm. Therefore, selecting this coupling distance allows the MRR to simultaneously achieve strong resonance characteristics and excellent sensing performance. Based on the above optimization results, the final structural parameters of the TSWG-MRR are summarized in Table 1. The optimized design provides a favorable balance among Q, ER, and *S_n_*, forming the basis for the subsequent sensing-performance analysis.

## 4. Sensing Effect

**Figure 10:**
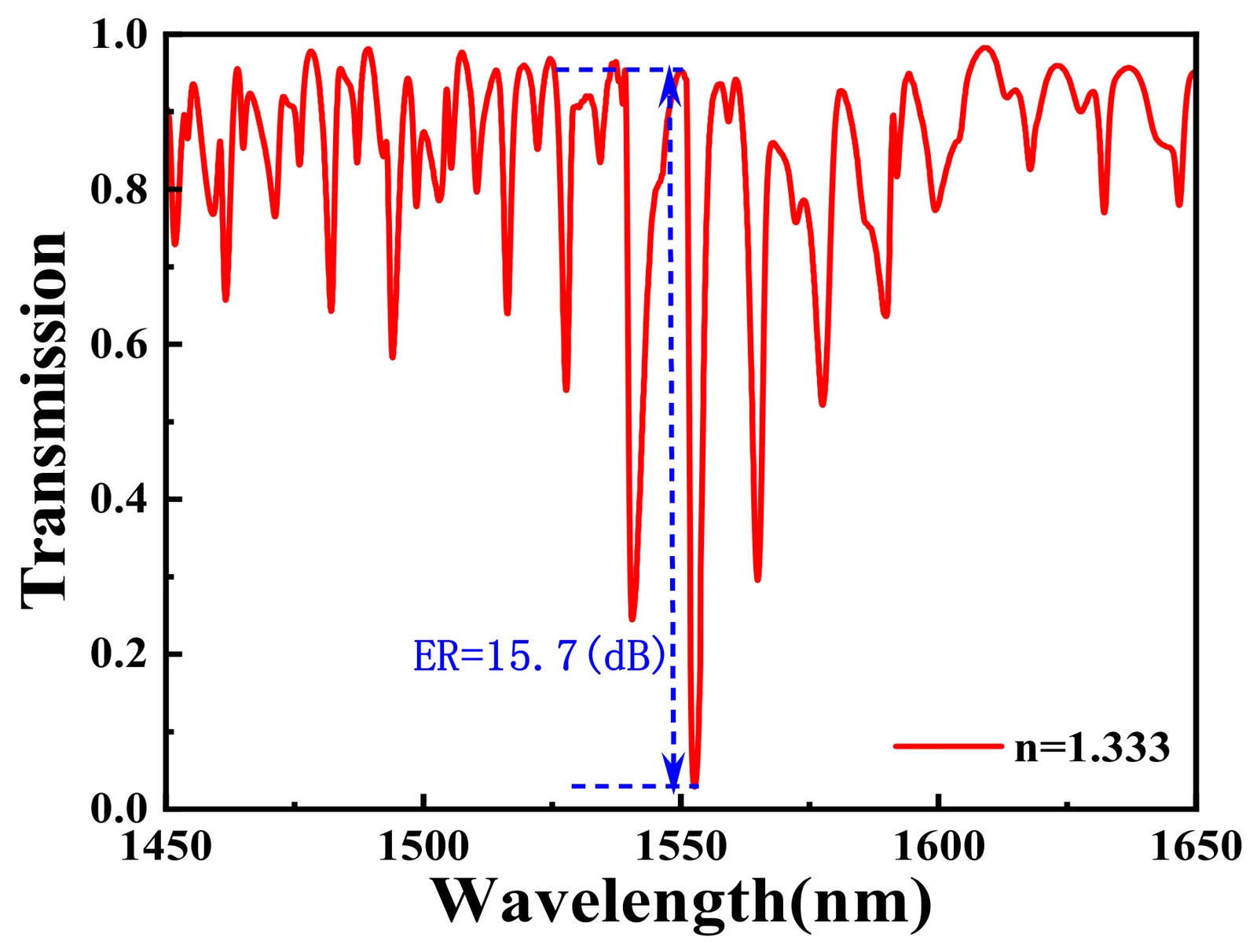
Transmission envelope spectrum of TSWG-MRR.

**Figure 11:**
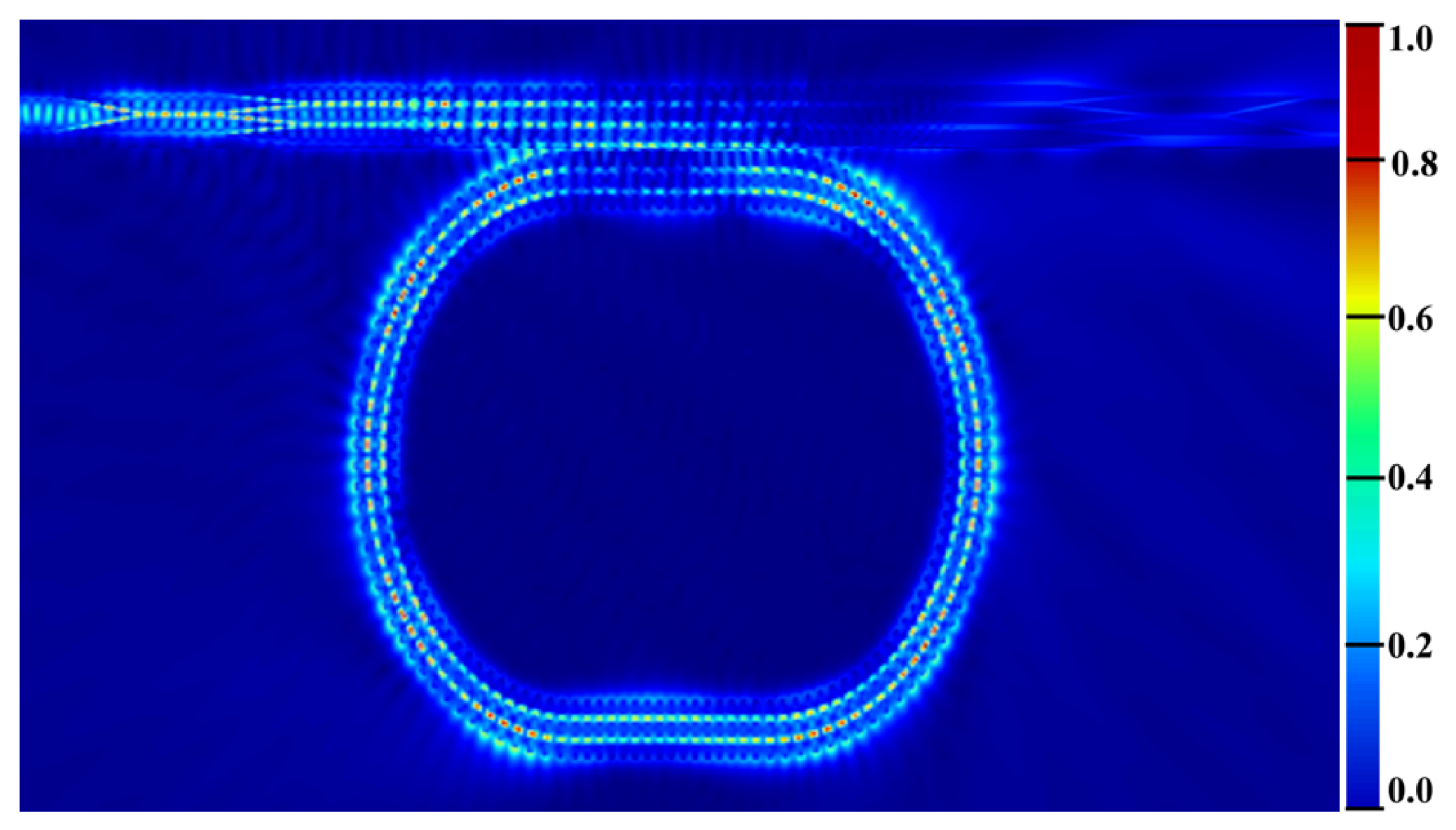
Power density distribution at the resonant peak of TSWG-MRR.

The normalized transmission spectrum of the optimized TSWG-MRR is shown in Figure 10. Unlike conventional micro-ring resonators that exhibit periodic resonance dips with nearly uniform amplitudes, the proposed device generates a distinct envelope-shaped transmission spectrum. An ER of 15.7 dB is achieved near the resonance wavelength of 1550 nm, indicating strong resonance modulation and efficient optical confinement. The formation of the envelope spectrum originates from the strong dispersive properties of the SWG waveguide. Owing to the periodic RI modulation introduced by the SWG structure, the propagating optical field is governed by Floquet–Bloch modes. Consequently, the resonance condition of the MRR becomes wavelength dependent, resulting in nonuniform resonance amplitudes across the transmission spectrum. This dispersion-induced modulation gradually shapes the resonance envelope observed in Figure 10. At the same time, multiple resonance peaks are generated by the constructive and destructive interference of optical fields circulating within the resonator. These resonance peaks are distributed beneath the slowly varying spectral envelope determined by the SWG dispersion characteristics. Therefore, the overall transmission spectrum can be regarded as a superposition of fine resonance oscillations and a broad envelope profile. Compared with conventional single-resonance tracking approaches, the envelope spectrum provides an additional spectral degree of freedom for sensing. By monitoring the global displacement of the envelope rather than a single resonance peak, the proposed sensing mechanism substantially expands the measurable RI range while maintaining high sensitivity. The electric-field distribution at the resonance wavelength is presented in Figure 11. A significant portion of the optical energy is concentrated within the slot regions and SWG structure, where strong evanescent fields interact directly with the surrounding analyte. This enhanced overlap between the optical mode and the sensing medium is primarily responsible for the high sensitivity achieved by the proposed device.

To further evaluate the practical applicability of the proposed device, calcium chloride (CaCl_2_) solutions with different concentrations were employed as the sensing medium in the numerical simulations Table 2 [32].

**Figure 12:**
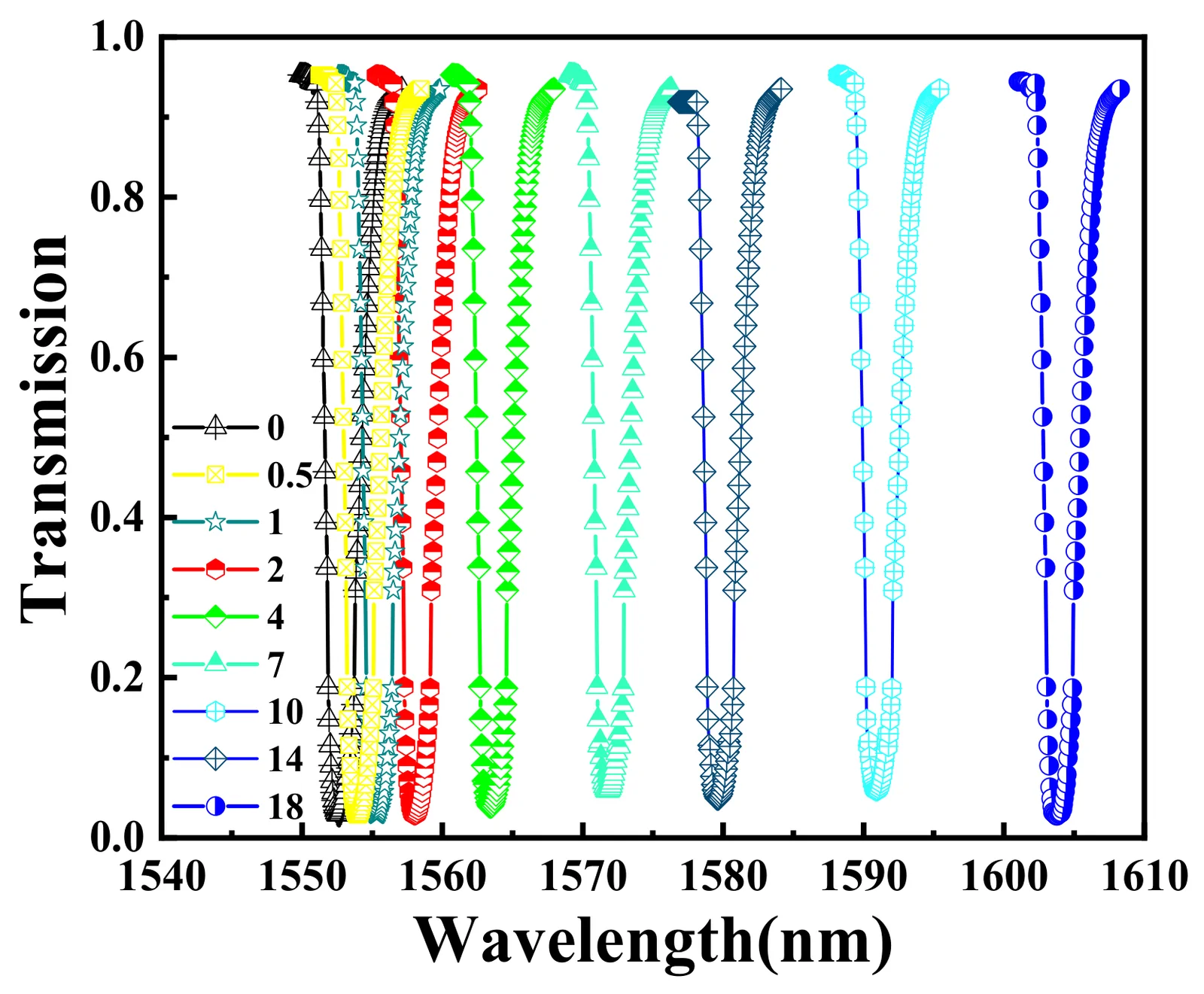
Monitoring the envelope-peak position of TSWG-MRR transmission spectra at different solution concentrations.

**Figure 13:**
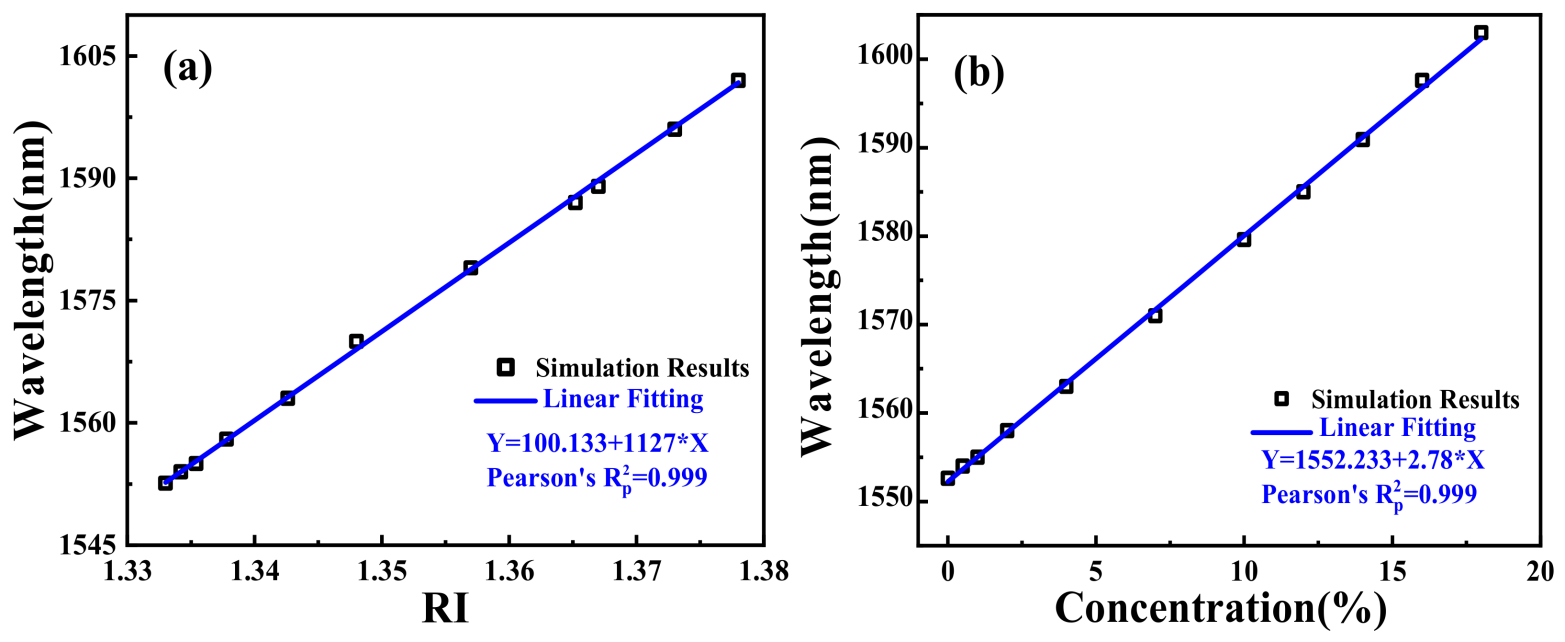
(**a**) RI sensitivity fitting curve of TSWG-MRR; (**b**) concentration sensitivity calibration curve of TSWG-MRR.

Changes in analyte concentration modify the refractive index of the surrounding medium, thereby altering the effective RI of the guided mode and inducing a measurable spectral shift. Figure 12 shows the envelope-peak positions extracted from the transmission spectra at different solution concentrations. It should be clarified that the tracked resonance dip is not an arbitrary peak, but the resonance at the envelope maximum (i.e., the deepest dip near 1550 nm, where the maximum ER of 15.7 dB is achieved). This choice is motivated by three considerations. First, the envelope maximum corresponds to the optimal coupling condition and hence the deepest resonance, which provides the highest signal-to-noise ratio and the most accurate wavelength extraction. Second, as the ambient RI increases, all resonance dips beneath the envelope redshift together with the envelope in a continuous and monotonic manner; meanwhile, the dips away from the envelope center become progressively shallower (i.e., their ER decreases), so their positions are less robustly identifiable. Third, because the envelope spectrum evolves continuously and smoothly with the RI without crossing resonance-order ambiguities, the envelope maximum can be uniquely identified throughout the entire sensing range of 0.45 RIU, in contrast to single-resonance tracking, which becomes ambiguous once the wavelength shift exceeds the FSR. To quantify the sensing performance, linear regression analysis was performed using the simulated data. The fitting results are presented in Figure 13a. A highly linear relationship is observed between RI variation and wavelength shift, demonstrating excellent sensing stability and predictability. The corresponding RI sensitivity is calculated to be 1127 nm/RIU. Figure 13b presents the concentration fit curve. A strong linear dependence is observed between calcium chloride concentration and wavelength shift, yielding a concentration sensitivity of 2780 pm/%. The high coefficient of determination confirms the reliability of the proposed sensing mechanism for quantitative concentration measurements. These results indicate that the proposed TSWG-MRR can serve as an effective platform for both RI sensing and concentration monitoring in biochemical and environmental applications.

The limit of detection (LOD) is a critical parameter for evaluating sensor performance. For wavelength-interrogated RI sensors, the LOD is defined as the minimum detectable RI variation and can be calculated using Equation (12).

(12) $$\mathrm{LOD}=\frac{\delta \lambda_{\min}}{S_{n}}$$

In Equation (12), Δ*λ_min_* represents the minimum resolvable wavelength shift of the measurement system, while *S_n_* denotes the RI sensitivity. Compared with conventional single-resonance tracking approaches, the envelope-based interrogation strategy improves spectral-tracking robustness over a broad RI range. Consequently, the proposed sensor maintains stable sensing performance even when large RI variations occur. Assuming a wavelength resolution of 10 pm and using the obtained sensitivity of 1127 nm/RIU, the theoretical detection limit is calculated to be 8.87 × 10^−6^ RIU. It should be noted that the envelope amplitude gradually decreases near the upper limit of the sensing range. This phenomenon may slightly reduce wavelength-extraction accuracy and therefore lead to a modest degradation in practical detection performance. Nevertheless, the calculated LOD remains highly competitive compared with previously reported SWG-based MRR.

To further demonstrate the advantages of the proposed design, its performance is compared with representative SWG-based micro-ring resonator sensors reported in the literature, as summarized in Table 3. Compared with previously reported devices, the proposed TSWG-MRR simultaneously achieves the highest sensitivity (1127 nm/RIU), the lowest theoretical detection limit (8.87 × 10^−6^ RIU), and the widest sensing range (0.45 RIU). Most importantly, the sensing range is more than four times larger than that of the best previously reported SWG-based MRR and exceeds the performance of conventional resonance-tracking schemes by more than one order of magnitude. These improvements are primarily attributed to the synergistic effects of the double-slot architecture, trapezoidal SWG configuration, and envelope-spectrum interrogation strategy. The double-slot structure enhances light–matter interaction, the trapezoidal geometry improves optical confinement and reduces scattering loss, and the envelope-tracking mechanism effectively removes the sensing-range limitation imposed by the FSR.

It should be noted that the Q of the proposed TSWG-MRR (1885) is lower than that of several reported SWG-MRRs listed in Table 3. This is an inherent trade-off of the double-slot and trapezoidal SWG design: the optical field is deliberately delocalized into the low-index slot and analyte regions to maximize the light-analyte overlap, which inevitably increases the optical loss and reduces the Q. Nevertheless, for a wavelength-interrogated refractive index, the limit of detection is determined by the ratio of the resolvable wavelength shift to the sensitivity rather than by the Q alone. Benefiting from the highest sensitivity of 1127 nm/RIU among the compared devices, the proposed sensor still achieves the lowest theoretical LOD of 8.87 × 10^−6^ RIU. Moreover, the reduced Q is still sufficient for unambiguous resonance tracking, as indicated by the ER of 15.7 dB. In wide-range, cost-effective lab-on-a-chip sensing scenarios, where an ultra-sharp resonance is not essential, the combination of an ultra-wide sensing range (0.45 RIU, more than one order of magnitude wider than the compared devices) and the highest sensitivity is more valuable than a high Q, which is typically accompanied by a narrow FSR and hence a limited measurement range.

**Figure 14:**
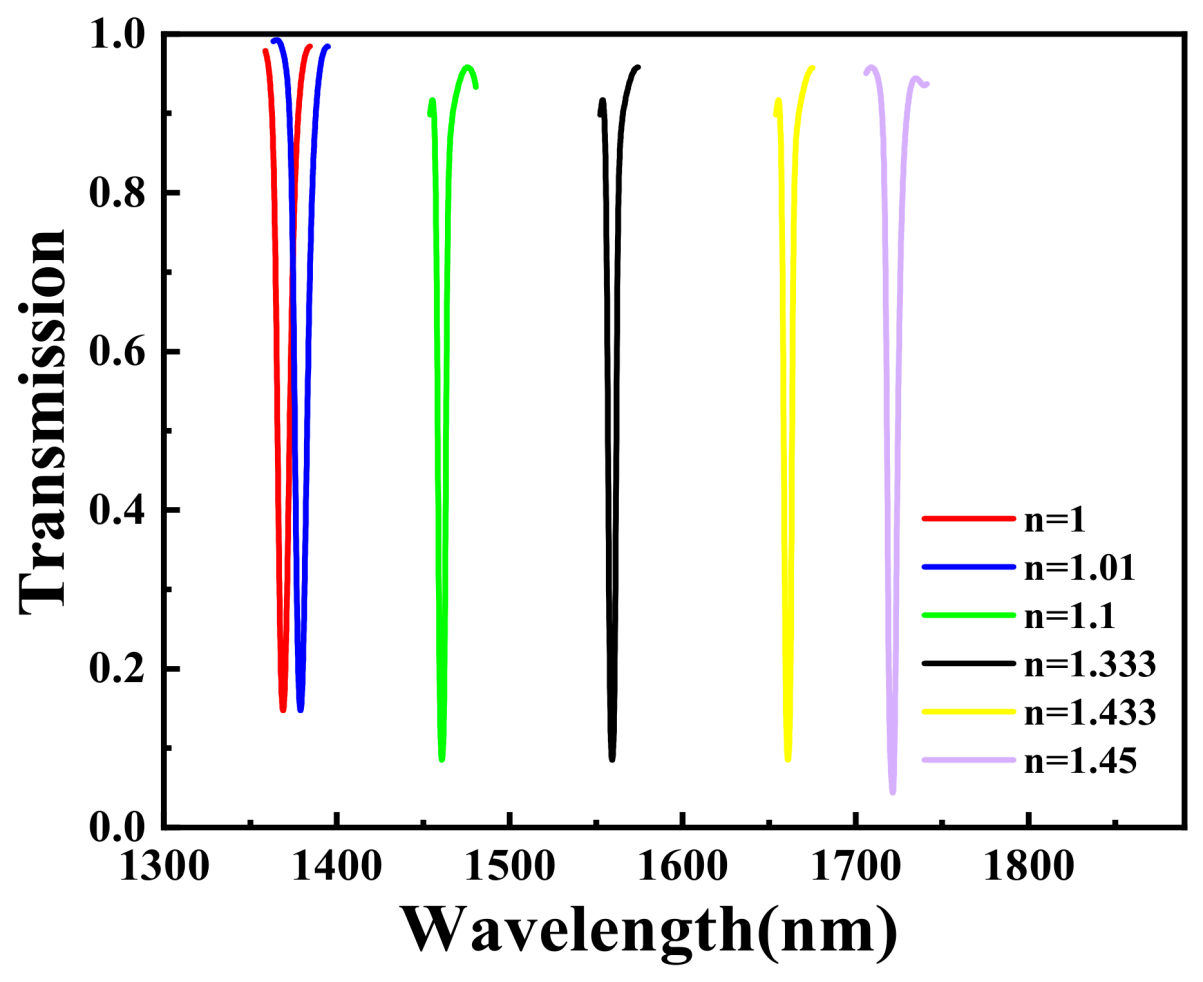
Envelope spectra under different cladding RIs.

To evaluate the sensing capability of the TSWG-MRR, simulations were performed under different cladding RI conditions. The corresponding transmission spectra are shown in Figure 14. As the surrounding RI increases, a pronounced redshift of the entire envelope spectrum can be observed. Unlike conventional MRR sensors that rely on tracking individual resonance peaks, the proposed approach utilizes the displacement of the envelope maximum as the sensing indicator [33,34,35]. This strategy significantly alleviates ambiguity caused by resonance-order transitions and effectively overcomes the sensing-range limitation imposed by the FSR. For gaseous sensing conditions, when the cladding RI increases from 1.00 to 1.01, the envelope peak shifts from 1369.4 nm to 1379.8 nm. Under liquid sensing conditions, the envelope peak moves from 1552.4 nm to 1665.1 nm as the RI increases from 1.333 to 1.433. The corresponding refractive-index sensitivity reaches 1127 nm/RIU. Furthermore, when the RI increases from 1.433 to 1.450, the envelope peak continues to shift from 1666.7 nm to 1721.5 nm. As a result, the measurable RI range is extended to 0.45 RIU. This value is substantially larger than those reported for conventional SWG-MRR sensors and demonstrates the effectiveness of the proposed envelope-spectrum tracking mechanism. The observed wide-range sensing capability arises from the fact that the envelope spectrum evolves continuously with RI variation.

Table 3 presents a comparison of the current SWG-MRRs.

**Table 3:** Comparison of sensing performance of existing SWG-MRRs.

Group	Device Structure	Material	LOD (RIU)	*S_n_* (nm/RIU)	Δn (RIU)
[20]	SWGRTR	Si	3.17 × 10^−4^	429.7	0.029 *
[21]	GDSWG	Si	8.15 × 10^−5^	760	0.1 *
[28]	SWGMR	Si	5.46 × 10^−5^	366	0.067 *
[36]	SWGS	Si	1.1 × 10^−4^	132	0.011 *
[37]	Multi-Box SWG-MRR	Si	1.02 × 10^−3^	579.5	0.012 *
[38]	T-SWGDSMRR	Si	3.3 × 10^−5^	424	0.02 *
[39]	SWG double-slot WG	Si	1.55 × 10^−4^	840	0.02 *
This paper	TSWG-MRR	Si	8.87 × 10^−6^	1127	0.45

* These data are extracted from the references and estimated based on the formula Δn = FSR/*S_n_*.

## 5. Conclusions

In this work, a novel TSWG-MRR is proposed for high-performance RI sensing. By integrating a double-slot waveguide architecture, trapezoidal subwavelength-grating structures, and an envelope-spectrum interrogation mechanism on an SOI platform, the proposed device effectively enhances light–matter interaction while simultaneously extending the measurable RI range beyond the limitation of conventional MRRs. Comprehensive numerical investigations based on the 2.5D FDTD method were conducted to optimize the key structural parameters of the device. In addition, a high-efficiency mode-conversion system consisting of STS, STDS, and DSTS converters was designed to ensure low-loss optical coupling between conventional strip waveguides and the SWG sensing region. The optimized mode converter achieves an average conversion efficiency exceeding 95% across the wavelength range of 1450–1650 nm, providing a robust foundation for high-performance sensing. The proposed TSWG-MRR exhibits a unique envelope-shaped transmission spectrum originating from the strong dispersive characteristics of the SWG structure. Consequently, the proposed TSWG-MRR successfully resolves the long-standing issue in integrated photonic sensors of the difficulty in simultaneously improving sensitivity and sensing range. Simulation results demonstrate that the optimized sensor achieves an RI sensitivity of 1127 nm/RIU, a concentration sensitivity of 2780 pm/%, a Q of 1885, and an extinction ratio of 15.7 dB. More importantly, the measurable RI range reaches up to 0.45 RIU, representing more than an order-of-magnitude improvement compared with conventional MRRs. Furthermore, the theoretical limit of detection is estimated to be 8.87 × 10^−6^ RIU, indicating excellent sensing resolution and competitive detection capability. A comparison with previously reported SWG-based resonator sensors demonstrates that the proposed TSWG-MRR simultaneously achieves high sensitivity, ultra-wide detection range, and a low detection limit. These improvements arise from the synergistic effects of the double-slot sensing architecture, trapezoidal SWG configuration, and envelope-spectrum interrogation strategy. Consequently, the proposed device effectively mitigates the long-standing trade-off between sensitivity and sensing range that commonly exists in integrated photonic sensors. In addition, the envelope-spectrum tracking concept may be extended to other resonant photonic devices, providing new opportunities for the development of high-performance integrated sensing platforms. Overall, the proposed TSWG-MRR offers a promising solution for next-generation label-free sensing applications and provides a new design paradigm for simultaneously achieving ultra-high sensitivity and an ultra-wide detection range in integrated photonic sensors.

## Figures and Tables

**Table 1:** Parameters of the TSWG-MRR resonator.

Symbol	Meaning	Value
FF	Fill factor	0.7 [nm]
Wslot	Slot width	100 [nm]
W	Width of the grating	300 [nm]
Wgap	Coupling distance	90 [nm]
Wtop	The top of the trapezoidal silicon block	155 [nm]
*Λ*	Grating period	300 [nm]
R	Radius of MRR	9.4 [µm]
L_s	Coupling length	17 [µm]
Hsio_2_	SiO_2_ layer height	2 [µm]
Height	Top silicon layer height	220 [nm]

**Table 2:** RI of a calcium chloride solution at 20 °C as a function of mass fraction.

Concentrations (%)	0	0.5	1.0	2.0	4.0	7.0	10.0	14.0	18.0
RI	1.333	1.334	1.335	1.337	1.342	1.350	1.357	1.367	1.378

## Data Availability

Data can be obtained from the authors upon reasonable request.
